# Co-creating a strategy for transforming person centred cultures

**DOI:** 10.3389/frhs.2025.1574632

**Published:** 2025-06-04

**Authors:** Karen Tuqiri, Suzanne Murray, Dan Shaw, Kate Hackett, Tanya McCance

**Affiliations:** ^1^ Nursing & Midwifery, South Eastern Syndey Local Health District, Sydney, NSW, Australia; ^2^ Prince of Wales Hospital, University of Technology, Sydney, NSW, Australia; ^3^ School of Nursing & Midwifery, University of Wollongong, Sydney, NSW, Australia; ^4^ School of Nursing and Paramedic Science, Institute of Nursing and Health Research, Ulster University, Belfast, United Kingdom; ^5^ Institute of Nursing and Health Research & Person-Centered Practice Research Centre, Queen Margarets University, Edinburgh, United Kingdom

**Keywords:** person-centred practice, nursing, midwifery strategy, co-creation, culture, leadership

## Abstract

**Introduction:**

Transforming healthcare systems to support person-centred practice reflects environments where individual values and beliefs are respected and where healthful cultures can flourish. However, there are significant challenges within healthcare systems that impact on the development of healthful workplace cultures. The nursing and midwifery professions need to play an influential role in formulating health policy and decision-making to contribute to health and social care systems that are underpinned by person-centredness. This paper reports the use of a practice development approach underpinned by the Person-Centred Practice Framework to co-create a strategy for nurses and midwives that will enable the development of person-centred healthcare practices. The key objectives are to demonstrate the processes that support co-creation to build consensus on what is strategically important to nurses and midwives; to gain an understanding of the value of external facilitation throughout the process and exploring the challenges encountered during the development of the strategy.

**Methods:**

Practice Development methodology was the approach used with skilled facilitation adopted to enable the working with values and beliefs, defining purpose and vision and establishing agreed working principles and behaviours. Consensus building methods were used to co-create draft strategy priorities further defined by wider stakeholder engagement.

**Results:**

A 5-year strategy was co-created with senior nursing and midwifery leaders, inclusive of key strategic priority areas and strategic actions. The seven priority areas align to the Person-Centred Practice Framework with underpinning shared purpose and values. (1) Developing Person-Centred Cultures, (2) Creating a Supportive Practice Environment, (3) Building Research Capacity, (4) Building a Dynamic Workforce, (5) Fostering Leadership at all Levels, (6) Enhancing Digital Informatics and New Technologies, (7) Delivering High Quality, (8) Safe Person-Centred Care. Together they provide a roadmap for implementation across the many nursing and midwifery contexts providing a solid foundation for leading and supporting person-centred practice across a large local health district with a focus on what matters most while continuing to be innovative in approaches to practice. The development of a clear shared purpose of person-centred practice and the exploration of values were critical first steps in the development of the strategy and provided a clear foundation from which the nursing and midwifery leaders could utilise for the ongoing strategic priorities and action discussions.

**Implications for practice:**

The development of nursing and midwifery strategy using Practice Development Methodology and the Person-centred Practice Framework enables critical dialogue that supports nursing and midwifery leaders identify key influences over nursing and midwifery practice. This approach not only fosters a sense of ownership and engagement among nurses and midwives but also ensures that their values, beliefs, and professional insights are integral to the strategic direction of healthcare practices. By aligning the strategy with the Person-Centred Practice Framework, nurses and midwives are better able to develop a shared understanding of person-centred practice where the individual needs and preferences of patients, families and staff are acknowledged. Overall, this strategy represents a significant step forward in supporting the professional development of nurses and midwives, enhancing the quality of patient care, and fostering a healthful culture where continuous improvement and innovation are at the forefront of the healthcare system.

## Introduction

Person-centredness is a global movement in healthcare simply because it reflects the importance of keeping people at the centre of healthcare systems (1, 2). It prioritises the human experience and places compassion, dignity and humanistic caring principles at the centre of planning and decision making and is translated through relationships that are built on effective interpersonal processes. We advocate the importance of the underpinning values of person-centredness, where the core value of “respect for the person” is paramount (3).

Transforming healthcare systems to support person-centred practice reflects environments where individual values and beliefs are respected and where healthful cultures can flourish. Healthful cultures are viewed as “contexts that are energy-giving for the benefit of health and wellbeing” (4). For healthful cultures to be achieved all persons need to be energised by the context in which they work and for that energy to connect with the personhood of all persons. This perspective on wellbeing ensures that person-centredness is not a uni-directional activity focusing on ensuring that service users have a good care experience at the expense of staff wellbeing.

Despite widespread acknowledgment that person-centredness is the appropriate underpinning philosophy for health and social care, person-centredness in practice is still misunderstood and difficult to operationalise and implement in practice. Whilst person-centredness permeates healthcare strategy and policy, the reality is that often stakeholders aren't actually talking about the same thing. We also see this dilemma in the published literature with interchangeable use of terms such as patient-centred and person-centred, leading to arguments that person-centredness is too difficult to define (5). Furthermore, in a recent editorial, McCormack (6) highlights the rhetoric of person-centred care often espoused in healthcare strategy and policy, but for many clinicians their lived experience of providing care and treatment on a daily basis can be very different.

Major healthcare external reviews internationally have identified that the contributions of key stakeholders are critical to improving the provision of quality health care and that the effectiveness of workplace culture is influenced by leadership (7–9). An important recommendation of the State of the World's Nursing report (10) is to strengthen nursing leadership to ensure that nurses play an influential role in formulating health policy and decision-making to contribute to effective health and social care systems. There is currently limited evidence identifying collaborative processes utilised by health care leaders in developing strategy (11). The Person-centred Practice Framework (3) identifies the need for strategic leadership within the macro level which informs the bringing together of nursing and midwifery leaders to explore current healthcare context and co-create strategy to influence practice and culture. Compassionate and person-centred leaders who embody a leadership approach that is considerate of each individual contribution, foster healthful relationships, invite, and encourage full engagement with the processes of decision-making and consensus making experience increased engagement, commitment, and trust across all levels of the organisation (12–14).

Within the Australian context, there is evidence of the impact of developing strategies that reflect innovation, creativity, and compassion in the way care is provided, and how supportive teams focus on individuals reaching their full potential (15, 16). This paper reports on an initiative that involved a senior group of nurse and midwifery leaders within a healthcare system within Australia who were committed and passionate about creating a strategic direction that would be enabling and supportive of the development of person-centred healthcare practices. It describes their engagement in collaborative processes to re-imagine a nursing and midwifery strategy through the lens of the Person-centred Practice Framework (3), ensuring a focus on what matters most to people across the system.

## Aims and objectives

### Aim

To explore the collaborative and inclusive process undertaken to co-create a strategy for Nursing and Midwifery that will support the development of person-centred healthcare practices.

### Objectives

• Demonstrate the processes that support co-creation to build consensus on what is strategically important to nurses and midwives
• To develop a strategy for transforming person-centered cultures in Nursing and Midwifery

### Methodology

The theoretical underpinnings for this initiative was the Person-centred Practice Framework developed by McCance et al. (3). This is a theoretical model developed from practice, for use in practice, which offers a unique perspective of person-centredness. The Framework has evolved over two decades of research and development activity and has made a significant contribution to the landscape of person-centredness globally. Not only does it enable the articulation of the dynamic nature of person-centredness, recognising complexity at different levels within healthcare systems, but it offers a common language and a shared understanding of person-centred practice. The Person-centred Practice Framework is underpinned by the following definition of person-centredness:

[A]n approach to practice established through the formation and fostering of healthful relationships between all care providers, service users and others significant to them in their lives. It is underpinned by values of respect for persons, individual right to self-determination, mutual respect and understanding. It is enabled by cultures of empowerment that foster continuous approaches to practice development (17).

**Figure 1:**
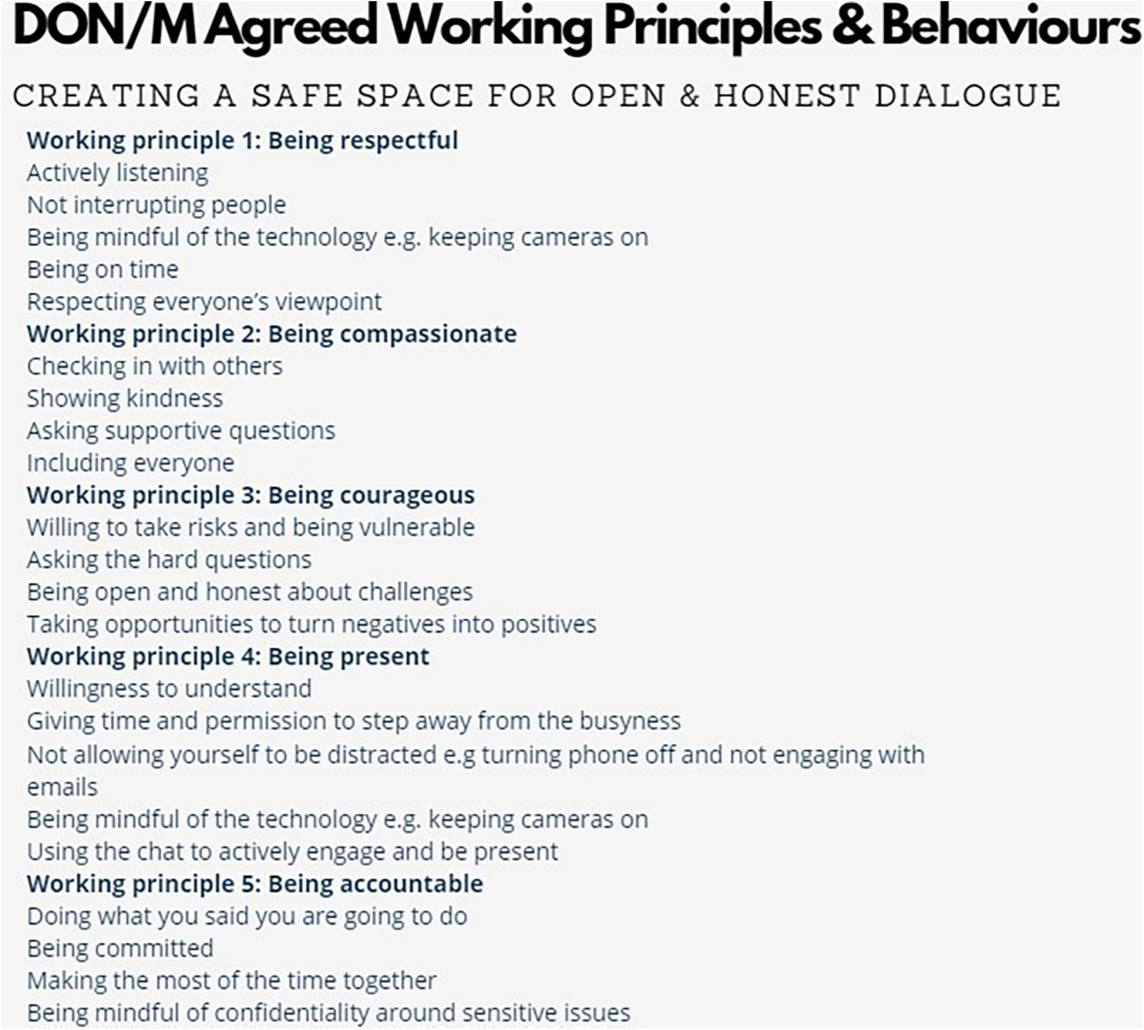
DON/M agreed working principles & behaviours.

The Person-centred Practice Framework comprises five domains: *prerequisites*, which focus on the attributes of staff; the *practice environment*, which focuses on the context in which healthcare is experienced; the *person-centred processes*, which focus on ways of engaging that are necessary to create connections between persons; and the *outcome*, which is the result of effective person-centred practice, that is a healthful; culture. Finally, these domains sit within the broader *macro context* (the fifth domain), reflecting the factors that are strategic and political in nature that influence the development of person-centred cultures. The relationships between the five constructs of the Person-centred Practice Framework are represented pictorially, that being, to reach the centre of the framework, one must first take account of the macro context, followed by consideration of the attributes of staff, as a prerequisite to managing the practice environment, in order to engage effectively through the person-centred processes, to bring about the outcome. The Person-centred Practice Framework is presented in Figure 1.

Practice development was the approach used and is a recognised methodology for enabling person-centred cultures (18, 19) which focus on supportive person-centred relationships and practice across individuals, teams, and systems to stimulate effective change and that are good places to work (20, 21). Practice Development (PD) includes the principles of collaboration, inclusiveness, and participation (CIP), active learning, skilled facilitation and is underpinned by shared values and purpose for person-centredness (19). A person-centred approach to facilitation was essential to engage the Directors of Nursing and Midwifery (DoNM) group across 12 sessions, this involved the approach of working with people rather than working on (22) and the skill of “being fluid”, agile in practice when necessary (23). Skilled facilitation is at the heart of PD as an enabler to developing an understanding of person-centredness, with facilitating the building of relationships within the DoNM group instrumental in them engaging individually and as a group (19, 22, 24). External skilled facilitators enabled the essential principles of CIP to be maintained throughout the strategy development process and the co-creation of a safe space to have critical discussion.

Co-creating working principles and behaviours enabled the DoNM group to openly share what a safe and supportive group space would look and feel like to them individually and then further develop as a collective group. This was facilitated virtually gaining consensus on how the group wanted to work collaboratively together in a respectful manner, these became the agreed “working principles and behaviours” and were also an important aspect of the group's relationship building (see Figure 1).

### Setting and sample

Located in New South Wales, Australia, the LHD is inclusive of nine hospitals, mental health and population and community health services, with approximately 5,000 nurses and midwives delivering health care to 930,000 residents. The LHD strategies align to the New South Wales Future Health Strategic Framework (2022–2032) vision and values, “A sustainable health system that delivers outcomes that matter most to patients and the community, is personalised, invests in wellness and is digitally enabled”, underpinned by the values of collaboration, openness, respect, and empowerment (25). Cultivating caring cultures that place people at the centre has been well established recognising the importance of treating each other as individuals, respecting personal beliefs, hopes and preferences, with the emphasis on kindness and what really matters to the person. The development of safe person-centred compassionate care has been embraced through engaging with the Heart of Caring Framework (26) that has guided the promotion of human-to-human connections, engaging effectively as teams, promoting self-care and wellbeing, and creating positive workplace cultures. Despite this focus the need for a co-created strategy inclusive of the nursing and midwifery workforce adopting a collaborative approach was identified. The LHD recognises skilled facilitation is at the core of enabling the development of person-centred cultures, continual investment in facilitation growth and development has been a LHD priority for over two decades.

Eleven Directors of Nursing and/or Midwifery co-created and led the strategy along with five members of the SESLHD Nursing and Midwifery Practice and Workforce Unit across over fifteen workshop sessions. For the engagement opportunities, the target population was Nurses and Midwives, working across the 8 public hospital including Population and Community Health and Mental Health Services within the LHD, with all role designations represented. Consumer participation was also included and considered important as a stakeholder group. Convenience sampling was used, with Nurses & Midwives invited to participate in the engagement opportunities. Thirteen Town Hall events were held with 390 Nurses and Midwives attending, with 2,127 items of feedback collected. Four Focus Groups were held with 65 attendees in total. All facilities and services from across the LHD were represented by their Director of Nursing and Midwifery and senior leaders. This led to a wider stakeholder engagement, which was critical to the strategy development to ensure collective ownership.

### Data collection & analysis

Data was collected throughout a range of approaches across the strategy development including values clarification and development of a shared purpose; identifying priorities areas through consensus; and wide stakeholder engagement.

#### Values clarification and shared purpose statement

Working with values and beliefs and defining purpose and vision, are key to person-centred practice. These processes have been identified as important for transforming workplace cultures and developing positive and effective working relationships with colleagues (17, 18, 27). Having a voice and being involved in decision making was fundamental, leading to on-going engagement, commitment and contribution of the DoNM group (21). The DoNM group first worked at an individual level and then collectively to co-create an agreed shared purpose and values statement. An exploration of purpose, values, and beliefs of “person centred practice” was undertaken, the external co-facilitation model enabled each DoNM to be actively involved in every aspect of the process. Individual reflection and responses were captured using a values clarification exercise (28), these were shared, collated, and themed in two smaller groups following critical dialogue, resulting in the co-creation of two draft purpose statements for “person centred practice”. Voting resulted in a preferred statement with further review of the second statement for any elements that needed to be integrated. Further facilitated dialogue enabled consensus on an agreed final purpose statement. Underpinning values were also considered, shared, and critically discussed. The DoNM group agreed that the previous LHD nursing and midwifery strategy “Journey to Care 2015–2020” six values should remain as they continue to be the values underpinning their shared purpose. An additional value “courage” as proposed and added with an accompanying value “in action” statement.

#### Identifying priorities by building consensus

Consensus building, also known as collaborative problem-solving or collaboration (29), is a process used to generate ideas, understand problems and to settle complex, multiparty issues. Building consensus was an intentional part of the facilitation to enable CIP principles and ensure each DoNM was part of the co-creation of the 5-year strategic plan, with a consensus of priority areas. Each DoNM prepared and had thoughtful consideration of their context and identified individual facility priorities, breaking into three groups to share, discuss further and collectively identify duplication and need for any rewording. Final priorities from each group were captured through an online visual work platform for collaboration. Facilitation enabled further consolidation and consensus building with seven key priorities being identified. The priorities were further refined and then mapped onto the Person-centred Practice Framework (3).

#### Engaging in stakeholder events

Feedback sessions in the form of online and face to face townhall events were agreed as a forum to engage Nurses and Midwives from across the LHD. These were opportunities for the facilitation team to engage in conversation with stakeholders, gaining their feedback on the purpose statement and strategic actions to achieve each of the seven priorities. We asked, “*what words speak to you/what do you connect with*” and “*what would be needed for this priority to be achieved?*”. A facilitator guide was developed to ensure consistency across events which included an opportunity to feedback on the draft purpose statement and values and the seven priority areas. The raw data obtained from stakeholder townhall events was reviewed. The data was analysed to answer the proposed question with each reviewer reading comments and organising into thematic categories for their allocated priority. Robust dialogue enabled the feedback data to be collated further and themed with draft strategic actions emerging for all seven priority areas along with priority descriptors of the focus and important.

The final part of the strategy development process involved virtual focus groups seeking feedback on the final draft strategy through the lens of nursing and midwifery leaders. Participants were asked “*What are the key messages you get from the strategy?*” “*How does the strategy resonate with you?*” and “*In reading the strategy, can you see yourself as a Nurse/Midwife within the strategy?*”

### Ethical considerations

The Human Research Ethics Committee (HREC), at the Low Negligible Risk review, approved the project. Participation in every aspect of the strategy development was voluntary with no coercion. All invites to participate at townhall events and focus groups followed ethical processes, ensuring and declaring anonymity throughout.

## Results

The following section presents results from the large data set collected across the development of the strategy. Table 1 summarises key data captured during the development of the strategy.

**Table 1:** Summary of engagement sessions.

Directors of nursing and midwifery group	15 facilitated sessions	
Townhall events	13 events and 390 attendees	2,127 items of feedback received
Focus Groups	4 focus groups and 65 attendees	

**Figure 2:**
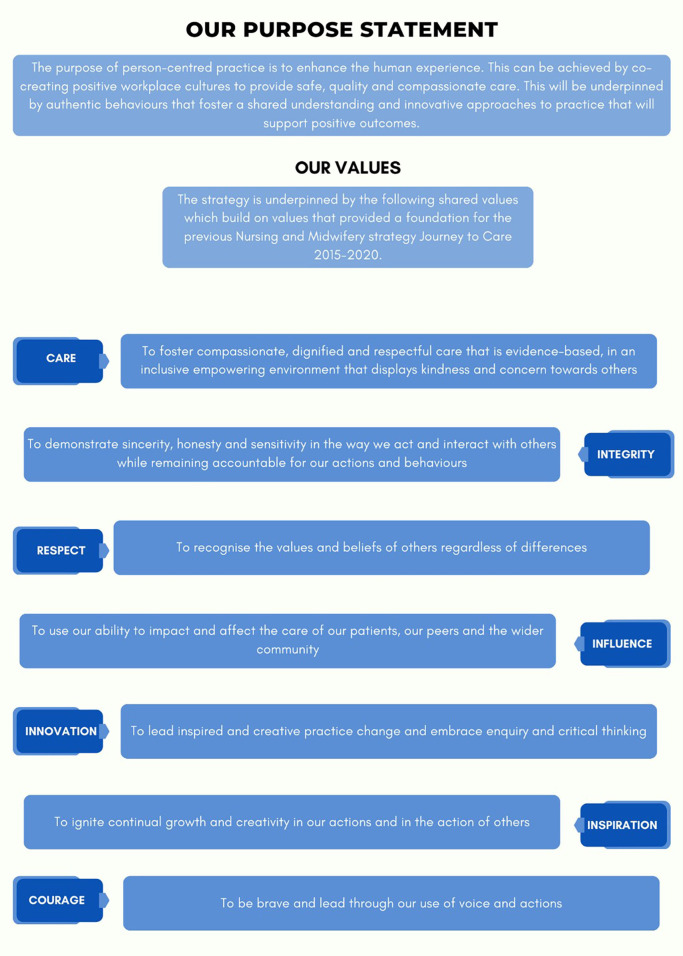
Our purpose statement and values.

The first outcome from this work was the co-creation of a shared purpose statement and values with person-centred practice at its core, which is presented in Figure 2. When asked in the peak Townhall event to review the purpose statement and share what words they connect with, our Nurses and Midwives identified that they connected most with “Authentic behaviours, co-creating, compassionate care, enhance the human experience, innovation, person-centred and positive workplace cultures” (Peak Townhall event).

One participant shared: “*I feel it (the purpose statement and values) aligns well with my own values, the purpose I feel is clear*” (Focus Group 4, Nurse Unit Managers and Midwifery Unit Managers). Another participant stated: “*I love the inclusion of courage as a value*”, “*It is values based and values people*”. (Focus Group 1, Nurse and Midwifery Managers).

A total of 17 priority areas were generated and then themed resulting in 6 key strategic priorities. This collaborative process was highly valued by the DoNMs: “*Using a shared online platform for collaboration enabled us as a group to bring along our own priorities and in a transparent and open forum, have a robust dialogue and work through a process of grouping, theming and coming to a consensus on our final priority areas*”. (Director of Nursing & Midwifery, Facilitated Session—Strategic Priority Consensus Building). A seventh priority area, Creating a Supportive Practice Environment, was identified through this process as it was noted in the facilitated session that the Practice Environment element of the PCPF was missing. The final Strategic Priority Areas are outlined in Table 2.

**Table 2:** Priority area consensus building.

Initial priority areas						
Flexible Approaches to Education, Patient Experience, Developing a Strong Nursing & Midwifery Voice, Staff Wellbeing, Professional Pathways, Recruitment and Retention, Meeting The Needs of Vulnerable Populations, Shared Understanding of Person-Centred Care, Preceptorship & Clinical Supervision, Effective Use of Resources, Communication for Positive Collaboration, Building/Developing Leadership, Digital and Informatics, Developing Person-Centred Culture, Building Capacity and Capability for Research, Capable Workforce, Flexible Approach to Education						
Secondary Priority Areas (Grouped)						
– Developing Person-Centred Culture – Shared Understanding of Person-Centred Care – Staff Wellbeing – Communication for Positive Collaboration – Meeting the Needs of Vulnerable Populations		– Quality & Safety – Patient Experience	– Building/Developing Leadership – Developing a Strong Nursing & Midwifery Voice	– Digital & Informatics	– Capable Workforce – Professional Pathways – Preceptorship & Clinical Supervision – Flexible Approaches to Education – Recruitment & Retention	– Building Capacity & Capability for Research
Final Priority Areas						
Developing Person-Centred Cultures	Creating a Supportive Practice Environment	Building Research Capacity	Building a Dynamic Workforce	Fostering Leadership at all Levels	Enhancing Digital Informatics and New Technologies	Delivering High Quality, Safe Person-Centred Care

**Figure 3:**
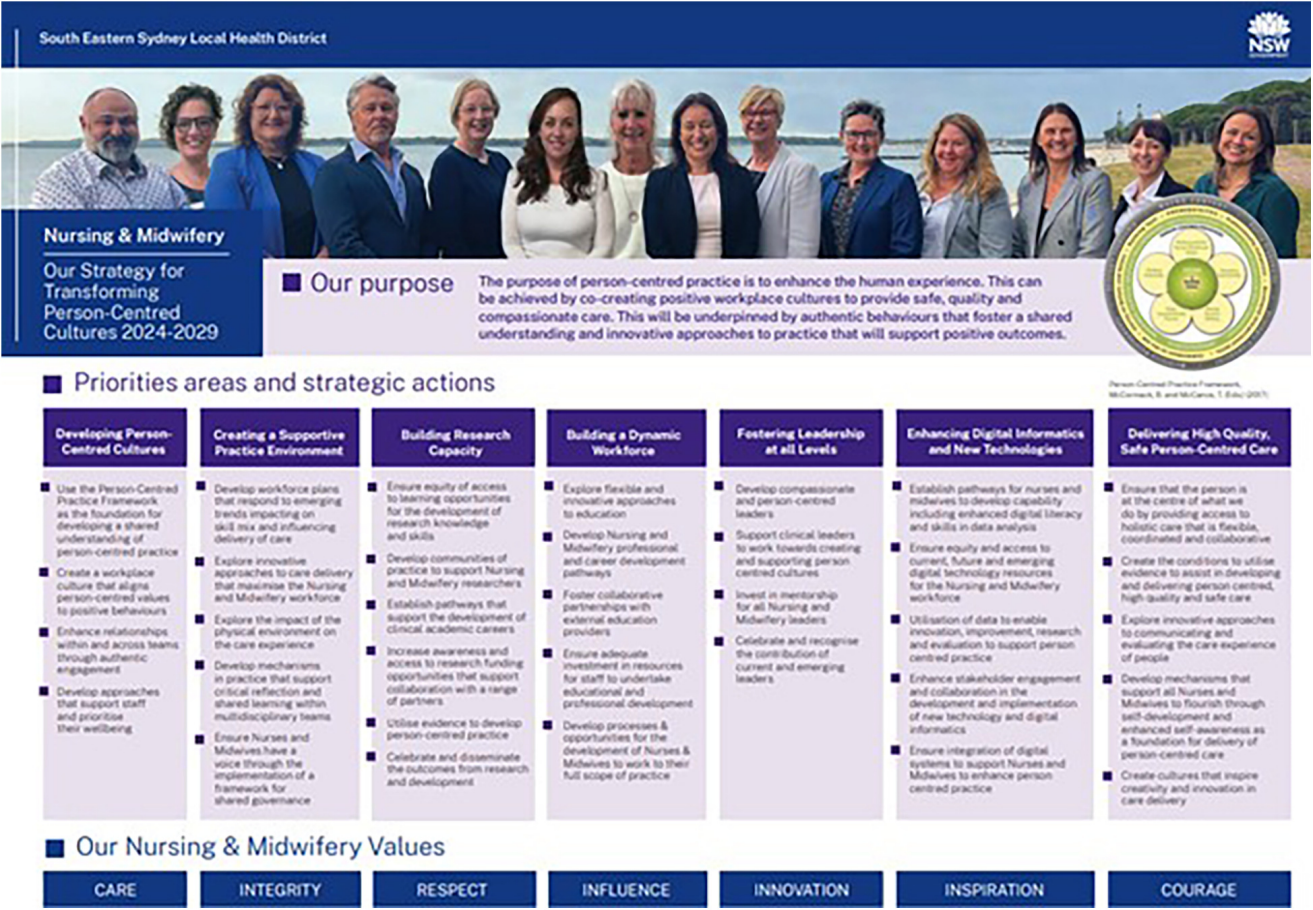
Alignment of strategic priorities to the person-centred practice framework.

During the Facilitated Session—Strategic Priority Consensus Building, it was identified that the seven final Strategic Priority Areas aligned with the Person-Centred Practice Framework. The seven final Strategic Priorities were then mapped to the Person-Centred Practice Framework to ensure there was a comprehensive approach to creating healthful workplace cultures as demonstrated in Figure 3.

The Strategic Priorities, shared with staff via the Town Hall events, garnered 1,406 suggestions for action areas. *Enhancing Digital Informatics and New Technologies* received the highest volume of feedback with 245 responses. The importance of nursing and midwifery having an active role in this rapidly evolving space was emphasised:

“Fair representation from all nursing and midwifery roles within the clinical councils that make big decisions on allocated resources to technology and recourses”, (Town Hall Event 3)

“Having a clear pathway for nurses and midwives to approach and follow for development of technologies in their clinical areas”, (Town Hall Event 2)

“Inclusion of nursing and midwifery in development of technology systems including during testing and planning for implementation” (Town Hall Event 6).

*Developing Person-Centred Cultures* received the least responses at 172. The sense that this was already happening in the organisation was evidenced form the data:

“Our weekly Multidisciplinary meeting structure and the way we work as a team with our patients, demonstrated through our daily interactions, shows that we are doing it” (Town Hall event 12).

However, there was also a sense that a consistent approach to developing person-centred practice would be valuable:

“Unpacking person-centred care will look different for each team, so a personalised and meaningful approach to doing so will be most effective” (Town Hall Event 9).

Strategic themes from the Townhall events are outlined in Table 3. Clear themes emerged from the collated dataset for each of the seven Priority Areas as presented in Table 3. These themes formed the basis for development of the strategic actions.

**Table 3:** Identified strategic themes from town Hall events.

Strategic priority	Responses	Themes	Agreed strategic actions
Developing Person-Centred Cultures	172	– Shared values and purpose – Staff having a voice – Team engagement – Wellbeing and positivity – Diversity	Use the Person-centred Practice Framework as the foundation for developing a shared understanding of person-centred practice. Create a workplace culture that aligns person-centred values to positive behaviours. Enhance relationships within and across teams through authentic engagement. Develop approaches that support staff and prioritise their wellbeing.
Creating a Supportive Environment	187	– Skill mix and staffing – Physical/clinical environment – Support, engagement and education – Staff and consumer experience – Models of care	Develop workforce plans that respond to emerging trends impacting skill mix and influencing delivery of care. Explore innovative approaches to care delivery that optimise the Nursing and Midwifery workforce. Explore the impact of the physical environment on the care experience. Develop mechanisms in practice that support critical reflection and shared learning within multidisciplinary teams. Ensure Nurses and Midwives have a voice through the implementation of a framework for shared governance.
Building Research Capacity	183	– Accessibility and resources – Education and support – Partnerships & collaboration – Governance – Professional development investment	Ensure equity of access to learning opportunities for the development of research knowledge and skills. Develop communities of practice to support Nursing and Midwifery researchers. Establish pathways that support the development of clinical academic careers. Increase awareness and access to research funding opportunities that support collaboration with a range of partners. Utilise evidence to develop person-centred practice. Celebrate and disseminate the outcomes from research and development.
Building a Dynamic Workforce	226	– Career development and pathways – Engagement and flexibility of workforce – Innovation – Partnerships – Recruitment processes – Resourcing – Mentoring & supervision – Succession planning	Explore flexible and innovative approaches to education Develop Nursing and Midwifery professional & career development pathways Foster collaborative partnerships with external education providers Ensure adequate investment in resources for staff to undertake educational and professional development Develop processes and opportunities for the development of Nurses and Midwives to work to their full scope of practice
Fostering Leadership at all levels	191	– Acknowledgement and appreciation – Leading culture – Leadership development – Empowerment – Mentoring – Pathways and programs – Reward and recognition – Succession planning	Develop compassionate & person-centred leaders Support clinical leaders to work towards creating & supporting person centred cultures Invest in mentorship for all nursing & midwifery leaders Celebrate and recognise the contribution of current & emerging leaders
Enhancing Digital Informatics and New Technologies	245	– Access to digital and technology resources – Digital and technology support and training – Using data – Stakeholder engagement and collaboration – Digital & Virtual Innovation and Research – Integrated systems	Establish pathways for Nurses and Midwives to develop capability including enhanced digital literacy and skills in data analysis Ensure equity and access to current, future and emerging digital technology resources for the Nursing and Midwifery workforce Utilisation of data to enable innovation, improvement, research and evaluation to support person centred practice Enhance stakeholder engagement and collaboration in the development and implementation of new technology and digital informatics Ensure integration of digital systems to support Nurses and Midwives to enhance person centred practice
Delivering high-quality safe person-centred care	202	– Consumer focus – Data access, evaluation and utilisation – Evidence Based Practice – Quality and safety – Resources – Training and education – Understanding self	Ensure that the person is at the centre of what we do by providing access to holistic care that is flexible, coordinated and collaborative Create the conditions to utilise evidence that assists in developing and delivering person-centred, high quality and safe care Explore innovative approaches to communicating and evaluating the care experience of people Develop mechanisms that support all Nurses and Midwives to flourish through self-development and enhanced self-awareness as a foundation for delivery of person-centred care Create cultures that inspire creativity and innovation in care delivery

Following the Town Hall events, the collated data was shared with key focus groups. When asked what their key messages were, Nursing & Midwifery Unit Managers shared “*The key messages I take-away relate to supporting nurses and midwives to deliver person centred care. Importantly, the context of care delivery has been explicitly acknowledged with the concept of healthful cultures and healthful relationships*”. In addition, they shared with us that the strategy resonated with them because, “*It has purpose, and the strategic actions are helpful to allow transfer to practice*”*.*

An early career Nurse shared with us that, “*This strategy clearly resonates with me as an Acting Clinical Nurse Educator. I believe that nursing workforce requires more support and environment where they can thrive and give their fullest*”*.*

Throughout all four focus groups, the question, “*In reading the strategy, can you see yourself as a Nurse and/or Midwife within the strategy*”, was posed. This was an important way to identify if the strategy truly represented the current workforce as well as providing a gauge for future engagement of Nurses and Midwives in delivering the strategy. A CNC highlighted for us that they, “*Can see myself as a nurse but also in my role, this framework will bring a consistent way for all Nurses and Midwives to align their practice and encourage increased collaboration*”.

Furthermore, our Nursing Unit Managers indicated that, “*The strategy encompasses all levels of health, can see myself from an RN to NUM across all of the priorities*”, “*Person centred care is widely used term and practice that is followed in patient care. The strategy provided will provide the support to nurses and midwives by supporting and developing skills in them, which help in practicing and achieving the person-centred care goals*”. Feedback from the focus groups on the priority areas and descriptors is outlined below in Table 4.

**Table 4:** Fous group feedback on priority areas and descriptors.

Priority Area	Descriptor	Feedback
Developing Person-Centred Cultures	Nurses and Midwives will develop a culture that evidences person-centred practice, is inclusive of all people, and is built on a shared understanding of their unique goals, expectations, wellbeing, and context. People and teams can personalise their experience and prioritise what really matters to them through relationships that are based on respect, integrity and trust.	*“A key message was the emphasis on the person-centred framework” & “What resonated with me was the strategic actions are helpful to allow transfer to practice”* (Focus Group 1 Nurse Managers). ‘*I liked the whole body of the strategic plan being based on the “Person-centred Practice Framework”. It makes a lot of sense to have that as the compass to drive all the actions that need to follow before we can achieve the goal of building “healthful cultures” across the district’* (Focus Group 2, Early Career Nurses & Midwives).
Creating a Supportive Environment	Nurses and Midwives will work collaboratively to create the conditions that support person-centred practice. This requires a focus on the development of shared decision-making processes that support effective staff relationships. It also requires an understanding of the impact of both skill mix and the physical environment on how Nurses and Midwives organise and deliver care.	*“This ensures that all staff have the tools and framework to work with persons in their treatment and recovery” & “This strategy clearly resonates with me as an Acting Clinical Nurse educator. I believe that nursing workforce requires more support and environment where they can thrive and give their fullest”.* (Focus Group 2, Early Career Nurses & Midwives)
Building Research Capacity	Nurses and Midwives will develop collective expertise in contributing to a sustainable research culture, enabling the generation and translation of knowledge that can support exceptional care. There is a need to continue to develop Nurses and Midwives to lead and support research initiatives that will shape clinical practice and workforce development. This will include participating in all types of research, often in collaboration with key partners.	*“This priority also is inclusive of enhancing staff skills, allow staff to be innovative in practice and quality initiatives and providing a culture that assists patients to be the centre of their care”.* (Focus Group 2, Early Career Nurses & Midwives)
Building a Dynamic Workforce	Nurses and Midwives will remain responsive, connected and engaged through focusing on innovative approaches required to develop Nursing and Midwifery careers and providing opportunities for staff to excel. This requires Nurses and Midwives to have learning resources that are easily accessible and appropriate, integrating both theory and practice. This will be enabled by working environments that are conducive to growth and development, supportive of the individual and team experience.	*“Recruitment and retention of nurses is dependent on positive culture environments. If this framework can be applied, it will have positive impacts on the patient care” (Focus Group 2, Early Career Nurses & Midwives)*. *“It focuses more on nursing and midwifery growth and development which is good”* (Focus Group 3, CNC/CMC). *“Building strong nursing workforce for future with capability and capacity”* (Focus Group 4, NUMs & MUMs).
Fostering Leadership at all Levels	Nurses and Midwives will lead, inspire and influence, regardless of the role and setting they work, in to create healthful cultures. This will be achieved by investing in more creative and effective ways of developing and supporting leaders. Nurses and Midwives will feel valued and respected and will have permission to lead and transform person-centred practice. This will be achieved by investing in a culture of recognition, developing a strong Nursing and Midwifery voice and acknowledging the achievement of our leaders.	*“The importance of strong leadership in facilities, the ongoing training and professional development opportunities to inspire staff to meet all of the priority areas”* (Focus Group 3, CNC/CMC). *“What resonates for me is that there is a focus on Leadership at all levels”* (Focus Group 4, NUMs & MUMs).
Enhancing Digital Informatics & New Technologies	Nurses and Midwives will embrace digital health, informatics and innovative technologies and incorporate these into clinical practice, whilst maintaining a focus on person-centred therapeutic relationships. This ensures Nurses and Midwives have the data and resources to continue to provide evidence-based, safe, quality, cost-effective and outcome-focused care for people into the future.	*“What resonates with me is the need to move with the times and the use technology by all staff”* (Focus Group 4, NUMs & MUMs).
Delivering High Quality, Safe Person-Centred Care	Nurses and Midwives will utilise the available evidence to evaluate their person-centred practice that is inclusive of all people and ensure there is a shared understanding of their unique goals, expectations, wellbeing, and context. People and teams can personalise their experience and prioritise what really matters to them, ensuring safe practice for all.	*“The strategy incorporates all aspects of care from the perspective of the patients” needs, but also provides a framework to assist staff in safe delivery of care in a positive health culture setting’* (Focus Group 2, Early Career Nurses & Midwives). *“Creates a positive shift to increase awareness of the patient experience”* (Focus Group 4, NUMs & MUMs).

**Figure 4:**
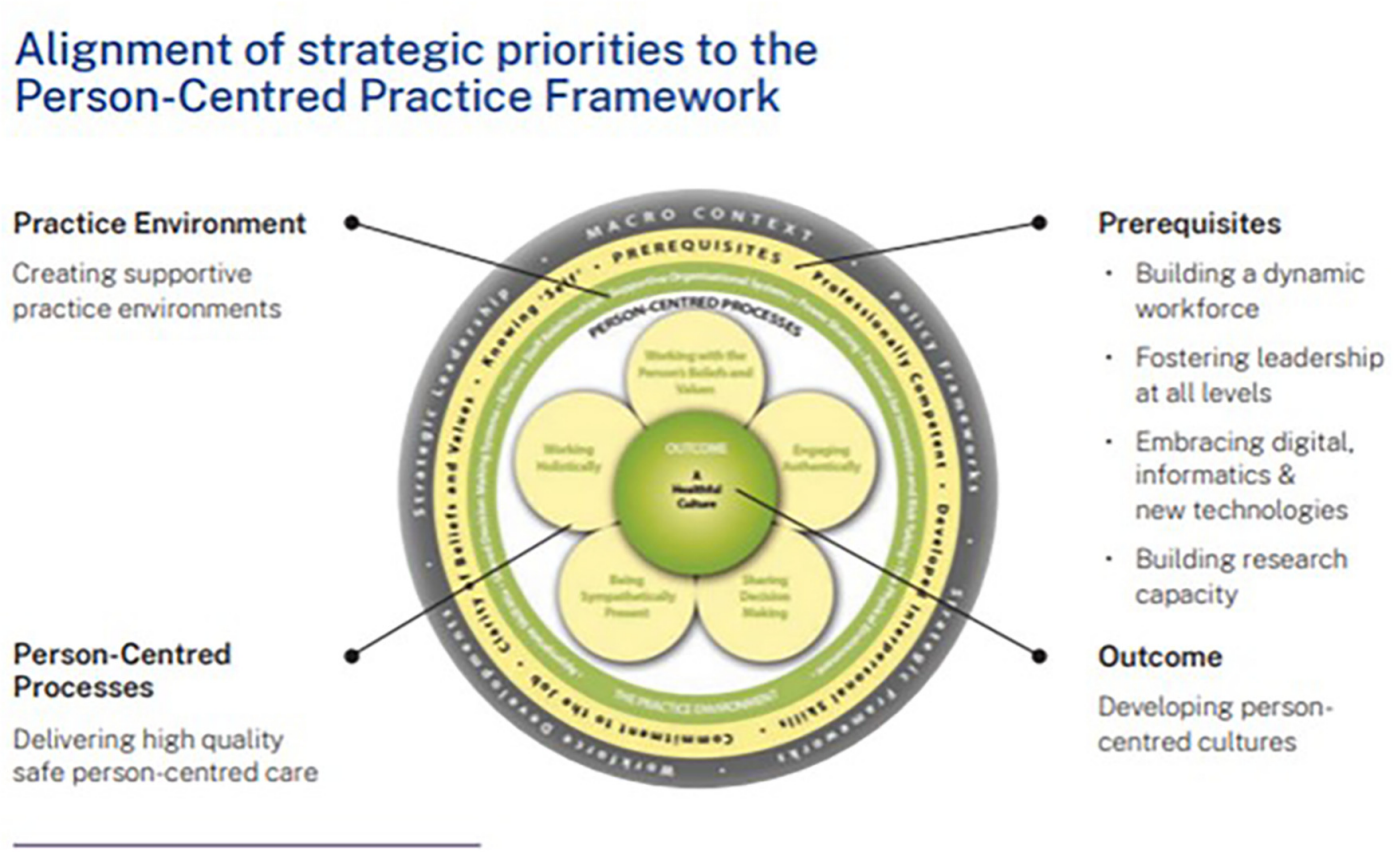
Priority and actions on a page. Reproduced with permission from “The Person-Centered Practice Framework” by Ailsa McMillan, Brendan McCormack, Cathy Bulley, Donna Brown, Suzanne Martin and Tanya McCance, licensed under CC BY 4.0.

There was an identified need to ensure the strategy was captured both in a comprehensive strategy document as well as in summary on a page to enhance usability and engagement. The final Priority Areas and Actions are outlined in Figure 4 below.

A key finding from the strategy development process was that using a mixture of both face to face and virtual skilled facilitation enabled the development of strong relationships, enhanced engagement between DoNMs, creating a high challenge and high support learning journey. Our DoNMs shared that, “it has been a fun and challenging journey to get to this point, external skilled facilitation had the role of taming us all and our thoughts and ideas whilst blending their [the facilitators] expertise and experience in person-centredness and challenging our thinking” and that “the facilitators encouraged us to just take a step back and think about what we wanted to do and where we wanted to take nursing and midwifery, the journey that we went on with our facilitators guided us in looking at how we can incorporate person-centred care into our practice here in our health district”.

The use of virtual skilled facilitation was effective for multiple aspects of the strategy development, with the exception of undertaking a Values Clarification Exercise (VCE) where the use of the virtual space challenged the ability to engage in the level of critical dialogue required. This was evident within the DoNMs shared experience where they stated that, “we had several sessions, I think there were twelve or so sessions in total as we went through, we tried to do some of the exercises with facilitation online, which definitely does not always work”. Switching to face-to-face skilled facilitation was essential to ensure that the VCE was undertaken effectively aligning to the CIP principles.

Relationship building between the DoNMs and their engagement throughout the strategy development was highlighted, they shared how “I found it a really good time to get to know our DoNM group at a closer level and also for us to get to know each other just a bit better, and that it was actually quite nice to be able to spend some time together in person”. Another DoNM stated how “it has been, from my perspective, really great working with the other DoNMs so closely and through this working with a DoNM who works in a very different environment (aged care) to where we work (maternity) further enhanced my experience”.

## Discussion

The development of nursing and midwifery strategy that acknowledged the multiple challenges of working within a large complex healthcare system including the social and political influences was important to ensure relevance, effectiveness and connection with our nurses and midwives. This program of work utilised the PCPF (3) as its foundation in which person-centredness and our current context could be explored. This enabled the DoNMs and other senior nursing and midwifery leaders to engage in critical discussion about their individual and collective understanding of what person-centredness meant in practice. Through exploring each domain of the PCPF and the current healthcare landscape in which we worked, a shared understanding of person-centred practice was achieved. This was an important first step in the strategy development as a shared understanding of what we were aiming to achieve through this strategy was essential. This is supported in the literature with the value placed on person-centredness increasingly being recognised in research and healthcare policy for its positive impact on outcomes for patients, staff and workplace cultures (21, 30, 33). Internationally, the World Health Organisation (WHO) (1) has identified the need for a shift in healthcare delivery which places the person at the centre of care, through the promotion of a comprehensive framework of people-centred health services. This exploration enabled the identification of key factors we would need to address to ensure we were able to develop and implement a strategy that supported person-centred practice and the transformation of culture. This is supported by McCance et al. (3) who highlights that whilst the knowledge base that underpins person-centredness has continued to expand an increased understanding of the multiple key elements that are required for effective implementation of person-centred healthcare practices is essential (3).

It is necessary that during the development of nursing and midwifery strategy, that aims to support person-centred practice and the development of healthful cultures, leaders identify and work with the influences on the diverse healthcare contexts in which nurses and midwives practice. This program of work aimed to develop a strategy that all nurses and midwives of all classifications could connect with. Following significant facilitated discussion and debate the priority areas of the leaders were explored and tested to fully understand their depth and content. The opportunity for nursing and midwifery leaders to explore and contextualise all influences on health care enabled the generation of strategic priority areas and actions that would support person-centred practice and were relevant and relatable to nurses and midwives across all levels of the broader organisation. The strategic priority areas identified can influence at the micro, meso and macro levels of healthcare organisations. The need to more fully understand and recognise the macro influences is captured within the PCPF and is identified as an essential component for the development of healthful cultures (3). Specifically, the important 5th domain of the PCPF—the macro context, acknowledges the need to understand the factors that are strategic and political in nature that will influence the development of person-centred cultures which include health and social care policy, strategic frameworks, workforce developments and strategic leadership (3).

This program of work recognised the importance of creating a shared vision and purpose statement of person-centred practice that would clearly articulate the direction and future within our organisation and could be utilised to connect with all nurses and midwives at all levels (31). The completion of a values clarification exercise to support the development of a shared purpose statement and the underlying values and behaviours provided a foundation for developing the strategy aimed at providing person-centred practice and transforming workplace culture. This is supported by Cardiff et al. (21) who identifies the need for strategic leadership to adopt a values-based approach and the development of a shared purpose where leaders can respectfully and constructively challenge each other while sharing the vision of what person-centred cultures look and feel like. The development of a clear shared purpose of person-centred practice and the exploration of values that underpin this purpose were critical first steps in the development of the strategy (32).

The creation of this shared understanding and values provided a clear foundation from which the nursing and midwifery leaders could utilise for the ongoing strategic priority and action discussions with all nurses and midwives. It is acknowledged that whilst nurses and midwives play a pivotal role in delivering person-centred practice at a microsystem level, the need for strategic leadership is a key factor in enabling person-centred cultures (3). The willingness of the DoNMs to be at times vulnerable, discuss what matters most to them professionally and identify with value-based behaviours that support person-centred practice was a significant component of the strategy development and represents authentic strategic leadership (38). It is necessary that this level of communication is supported to enable the development of trust and relationships within senior leadership groups. It is well documented that leaders who can adopt a value-based approach, are considerate of individual contributions, foster shared- decision-making whilst maintaining healthful relationships experience positive outcomes such as increased engagement with staff, commitment and trust across all levels of the organisation and improved staff outcomes (9, 12, 34). These outcomes are all are important considerations of successful strategy implementation.

The DoNMs recognised that person-centred cultures cannot be achieved by individuals alone and involvement of all key stakeholders was necessary throughout the process. To support the engagement of all stakeholders skilled facilitation using a collaborative, inclusive, and participative process (using CIP principles) was adopted. The use of CIP is a core foundation of practice development which offers a framework for implementing, monitoring and enhancing effective, evidence-based strategies that aim to achieve systems-wide sustainable change (20, 22). The co-creation of the shared purpose and values encouraged engagement and ownership from the DoNM group of the strategy, and the building of person-centred relationships. The commitment to a collaborative and inclusive way of working ensuring all nursing and midwifery staff had an opportunity to contribute further demonstrated a willingness to embrace a co-production model. The use of co-production models within healthcare research, policy development and education have been well established (27, 35). Co-production models have a range of advantages such as enabling stakeholders to have a voice as well as equalising power among users, clinicians and leaders (36). Oye et al. (37) broadly outline how the use of practice development principles utilised to support person-centred practice and the transformation of workplace cultures which have people at their centre.

Skilled facilitation, supported by a knowledge of co-production and use of person-centred approaches were essential aspects of engaging the DoNMs and staff who attended the stakeholder forums. Staff perceptions toward the organisation's commitment to its values, priority areas and direction were enhanced using skilled facilitation to elicit critical discussion with stakeholders on the strategy for nursing and midwifery into the future. This critical discussion enabled critique, alternative options and the development of a shared language that would connect with nurses and midwives at all levels within the organisation. This is reaffirmed by Oye et al. (37) who highlights the importance of using facilitation as critical to enabling reflection and practical consideration of how elements of the strategy impact on nurses and midwives as well as those receiving care at the individual, team, organisation and system levels.

## Conclusion

Developing strategy through the lens of the Person-Centred Practice Framework, skilled facilitation and the use of CIP principles for co-creation has resulted in the establishment of our 5-year strategy. This strategy provides a solid foundation for leading and supporting nurses and midwives to reach their potential, focus on what matters most and continue to be innovative in approaches to practice. The strategy provides a clear direction for enabling the development of healthful cultures that enable human flourishing for those who give care and those who receive care.

## Data Availability

The raw data supporting the conclusions of this article will be made available by the authors, without undue reservation.

## References

[1] World Health Organization. WHO Global Strategy on Integrated People-Centred Health Services. Geneva: World Health Organization (2015). Available at: https://apps.who.int/iris/bitstream/handle/10665/155002/WHO_HIS_SDS_2015.6_eng.pdf;jsessionid=EFD32B7F91380E03954F2F500CFEF280?sequence=1 (Accessed September 12, 2024).

[2] Nolte E Merkur S Anell A. Person centredness: exploring its evolution and meaning in the health system context. Chapter 2. In: Nolte E, editor. Achieving Person-Centred Health Systems: Evidence, Strategies and Challenges. Cambridge, UK: Cambridge University Press (2020). p. 19–38.

[3] McCormack B Dewing J McCance T. Person-centred Nursing Research: Methodology, Methods and Outcomes. Cham: Springer (2021).

[4] McCance T Brown D McCormack B Bulley C McMillan A Martin S. (Eds.). Fundamentals of Person-centred healthcare Practice. Oxford: John Wiley & Sons (2021).

[5] Mitchell P Cribb A Entwistle V. Vagueness and variety in person-centred care. Wellcome Open Res. (2022) 7:170. 10.12688/wellcomeopenres.17970.1 35865218 PMC9277200

[6] McCormack B. Person and family centredness—the need for clarity of focus. Eur Burn J. (2024) 5:166–8. 10.3390/ebj5020014 39599986 PMC11544900

[7] NSW Government. (2018). State of the NSW Public Sector Report 2018. Sydney, NSW: NSW Public Service Commission.

[8] Garling P. Final report of the Special Commission of Inquiry: acute care services in NSW public hospitals. State of New South Wales. (2008). Available at: https://www.cec.health.nsw.gov.au/__data/assets/pdf_file/0011/258698/Garling-Inquiry.pdf (Accessed November 22, 2025).

[9] Francis R. Report of the Mid Staffordshire NHS Foundation Trust Public Inquiry. London: The Stationery Office (2013). Available at: https://www.gov.uk/government/publications/report-of-the-mid-staffordshire-nhs-foundation-trust-public-inquiry (Accessed July 11, 2024).

[10] World Health Organization. State of the World’s Nursing Report—sOWN. Geneva: World Health Organization (2020). Available at: https://www.who.int/ publications/i/item/9789240003279 (Accessed January 23, 2024).

[11] Fulop N Ramsay A. How organisations contribute to improving the quality of healthcare. Br Med J. (2019) 365:l1773. 10.1136/bmj.l1773 31048322 PMC6495298

[12] West M. Compassionate Leadership, Sustaining Wisdom, Humanity and Presence in Health and Social Care. UK: The Swirling Leaf Press (2021).

[13] Cardiff S McCormack B McCance T. Person-centred leadership: a relational approach to leadership derived through action research. J Clin Nurs. (2018) 27(15-16):3056–69. 29679402 10.1111/jocn.14492

[14] Grover S Manville C Adib-Dupont MA Hasel M. Trust recovery between leaders and followers: the importance of character attributions. Acad Manag. (2015) 2015(1):10695–10695. 10.5465/AMBPP.2015.10695

[15] Schwarts S. Educating the Nurse of the Future—report of the Independent Review into Nursing Education. Canberra: Commonwealth of Australia (2019). p. 2019. Available at: https://www.health.gov.au/sites/default/files/documents/2019/12/educating-the-nurse-of-the-future.pdf (Accessed November 4, 2024).

[16] Australian Government Department of Health and Aged Care. (2024). Consultation and research summary report Building the evidence base for a National Nursing Workforce Strategy. Available at: https://www.health.gov.au/sites/default/files/2024-05/national-nursing-workforce-strategy-consultation-and-research-summary-report.pdf (Accessed November 4, 2024).

[17] McCormack B Manley K Titchen A. Practice Development in Nursing and Healthcare. Chichester: Wiley-Blackwell (2013).

[18] McCormack B McCance T. Person-centred Practice in Nursing and Health Care: Theory and Practice. 2nd edn Oxford: Wiley Blackwell (2017).

[19] Manley K Wilson V Oye C. Transforming health & social care using practice development. In: Manley K Wilson V Oye C, editors. International Practice Development in Health and Social Care. 2nd ed. New Jersey, USA: Wiley-Blackwell) (2021). p. 1–13.

[20] Hardy S Clarke V Frei IA Morley C Odell J White C A global manifesto for practice development: revisiting core principles. In: Manley K Wilson V Oye C, editors. in International Practice Development in Health and Social Care. 2nd ed. New Jersey, USA: Wiley-Blackwell (2021). p. 1–13.

[21] Cardiff S Sanders K Webster J Manley K. Guiding lights for effective workplace cultures that are also good places to work. Int Pract Dev J. (2020) 10(2):1–20. 10.19043/ipdj.102.002

[22] Middleton R Kelly M Dickson C Wilson V Lieshout F Hirter K Unpacking and developing facilitation. In: Manley K Wilson V Oye C, editors. International Practice Development in Health and Social Care. 2nd ed. New Jersey, USA: Wiley-Blackwell (2021). p. 131–46.

[23] Kelly M. Skilled Facilitation Within Transformational Practice Development in Healthcare. [PhD Thesis]. Sydney (NSW): University of Technology (2018).

[24] Crisp J Wilson V. How do facilitators of practice development gain the expertise required to support vital transformation of practice and workplace cultures? Nurse Educ Pract. (2011) 11(3):173–8. 10.1016/j.nepr.2010.08.005 20829114

[25] NSW Ministry of Health. (2022). Future health: guiding the next decade of care in NSW 2022–2032. Available at: https://www.frontiersin.org/journals/health-services/for-authors/author-guidelines (Accessed July 12, 2023).

[26] Murray S Tuqiri K. The heart of caring- understanding compassionate care through storytelling. Int Pract Dev J. (2020) 1:4. 10.19043/ipdj.101.004

[27] Lynch B Barron D McKinlay L. Connecting with others. In: McCormack B McCance T Bulley C Brown D McMillan A Martin S, editors. Fundamentals of Person-Centred Healthcare Practice. Oxford: Wiley-Blackwell) (2021). p. 93–101.

[28] Warfield C Manley K. Developing a new philosophy in the NDU. Nurs Stand. (1990) 4:41. 10.7748/ns.4.41.27.s37 2116867

[29] Burgess H Spangler B. Consensus Building. Intractable Conflict Knowledge Base Project Conflict Research Consortium. Boulder, CO: University of Colorado (2003). http://www.beyondintractability.org/m/consensus_building.jsp (Accessed August 23, 2024).

[30] Australian Commission on Safety and Quality in Health Care. (2018). Review of the key attributes of high-performing person-centred healthcare organisations. Available at: https://www.safetyandquality.gov.au/sites/default/files/migrated/FINAL-REPORT-Attributes-of-person-centred-healthcare-organisations-2018.pdf (Accessed January 21, 2025).

[31] Martin J McCormack B Fitzsimons D Spirig R. The importance of inspiring a shared vision. Int Pract Dev J. (2014) 2:4. 10.19043/ipdj.42.004

[32] Kouzes J Posner B. The Leadership Challenge: How to Make Extraordinary Things Happen in Organizations. 7th ed. New Jersey: John Wiley & Sons (2023).

[33] Klancnik Gruden M Turk E McCormack B Stiglic G. Impact of person-centered interventions on patient outcomes in acute care settings: a systematic review. J Nurs Care Qual. (2021) 36:1. 10.1097/NCQ.0000000000000471 32032336

[34] Alilyyani B Wong CA Cummings G. Antecedents, mediators, and outcomes of authentic leadership in healthcare: a systematic review. Int J Nurs Stud. (2018) 7:83. 10.1016/j.ijnurstu.2018.04.001 29684833

[35] O'Connor S Zhang M Kovach Trout K Snibsoer AK. Co-production in nursing and midwifery education: a systematic review of the literature. Nurse Educ Today. (2021) 7:102. 10.1016/j.nedt.2021.104900 33905899

[36] Makey M Walsh L Salih I. Co-production: what it is and how it can ensure inclusive practice for service users and staff. Nurs Manage J. (2023) 30:1. 10.7748/nm.2022.e2046 35818798

[37] Oye C Wilson V Manley K. Practice development—towards co-creation, innovation and systems transformation to foster person-centred care. In: Manley K Wilson V Oye C, editors. International Practice Development in Health and Social Care. 2nd ed. New Jersey, USA: Wiley-Blackwell (2021). p. 1–13.

[38] West MA Lyubovnikova J Eckert R Denis JL. Collective leadership for cultures of high-quality healthcare. J Organ Eff People Perform. (2014) 1:3. 10.1108/JOEPP-07-2014-0039

